# A phase 1 study evaluating the pharmacokinetics and preliminary efficacy of veliparib (ABT-888) in combination with carboplatin/paclitaxel in Japanese subjects with non-small cell lung cancer (NSCLC)

**DOI:** 10.1007/s00280-015-2876-7

**Published:** 2015-10-03

**Authors:** Hidenori Mizugaki, Noboru Yamamoto, Hiroshi Nokihara, Yutaka Fujiwara, Hidehito Horinouchi, Shintaro Kanda, Satoru Kitazono, Shigehiro Yagishita, Hao Xiong, Jane Qian, Hideyuki Hashiba, Stacie P. Shepherd, Vincent Giranda, Tomohide Tamura

**Affiliations:** Department of Thoracic Oncology, National Cancer Center Hospital, Tsukiji 5-1-1, Chuo-ku, Tokyo, 104-0045 Japan; AbbVie, Inc., North Chicago, IL USA; AbbVie, GK, Tokyo, Japan

**Keywords:** Veliparib (ABT-888), NSCLC, PARP, Carboplatin, Paclitaxel

## Abstract

**Introduction:**

Veliparib is a potent, orally bioavailable PARP inhibitor that enhances efficacy of DNA-damaging chemotherapeutic agents. The study objectives were to determine the recommended phase 2 dose (RPTD) of veliparib plus carboplatin and paclitaxel, and assess pharmacokinetics (PK), tolerability, and preliminary efficacy in Japanese patients with solid tumors.

**Methods:**

Carboplatin (AUC 6 mg/mL min) and paclitaxel (200 mg/m^2^) were administered on day 3 of a 21-day cycle. Oral veliparib (40, 80, or 120 mg BID) was administered on days 1–7. Patients received ≤6 cycles. Adverse events (AEs) were reported using NCI-CTCAE version 4.03, PK parameters were analyzed using noncompartmental methods, and responses were measured by RECIST version 1.1.

**Results:**

Twelve patients with non-small cell lung cancer (NSCLC) were treated. Common treatment-emergent AEs, consistent with toxicities associated with carboplatin and paclitaxel, included leukopenia (100 %), neutropenia (100 %), anemia (83 %), thrombocytopenia (75 %), increased alanine aminotransferase (67 %), and increased aspartate aminotransferase (67 %). Grade 3/4 AEs (in ≥2 patients) included neutropenia (100 %), leukopenia (33 %), anemia (25 %), and hyponatremia (17 %). No AEs led to veliparib, carboplatin, or paclitaxel interruption; no DLTs were observed. The RPTD was determined to be 120 mg BID. Veliparib *C*_max_ and AUC were approximately dose proportional. Six partial responses were observed.

**Conclusions:**

Veliparib PK was not impacted by carboplatin and paclitaxel. The safety profile was manageable. The 120 mg BID RPTD confirmed in Japanese patients is the dose being evaluated in global studies of veliparib. Preliminary efficacy suggests veliparib may enhance carboplatin and paclitaxel activity, providing benefit to patients with NSCLC.

## Introduction

Lung cancer is the most common cancer worldwide, with approximately 1.8 million new cases diagnosed globally in 2012 [1]. Patients with metastatic non-small cell lung cancer (NSCLC) are often diagnosed at a late stage and have a poor prognosis.

Platinum-based chemotherapy regimens, including the combination of carboplatin and paclitaxel, remain the current standard of care for patients with NSCLC [2–4]. Improving the efficacy of these regimens is an important priority to achieve more active and individualized treatment options and improve patient outcomes. Targeted therapies are emerging for the treatment of NSCLC, including epidermal growth factor receptor (EGFR) inhibitors, vascular endothelial growth factor receptor (VEGF) inhibitors, and checkpoint inhibitors that target the programmed cell death protein 1 (PD-1) pathway [2, 5]. There has been particular interest in agents that can be safely combined with commonly used chemotherapy regimens; the VEGF inhibitor bevacizumab has been recommended for the use with carboplatin and paclitaxel [2]. Inhibitors of poly (ADP-ribose) polymerase (PARP) represent another class of novel agents with potential utility when given with the standard of care chemotherapy.

Dysregulation of DNA repair has been associated with resistance to platinum-based therapies in patients with NSCLC and can negatively impact survival [6, 7]. PARP-1 and PARP-2 are nuclear enzymes that recognize DNA damage and facilitate DNA damage repair [8, 9]. PARP inhibition leads to unrepaired DNA damage, accumulation of platinum-DNA adducts, and increased death of cancer cells [10, 11]. When given with platinum-based chemotherapy, PARP inhibitors may improve outcomes in patients with NSCLC.

Veliparib is a potent, orally bioavailable, selective inhibitor of PARP-1 and PARP-2, with in vitro K_i_ values of 5.2 and 2.9 nM, respectively [10, 12]. In preclinical lung cancer tumor models, veliparib potentiates the effect of platinum-based chemotherapy [10, 13].

Veliparib has been safely combined with standard doses of carboplatin and paclitaxel in early phase studies in patients with advanced solid tumors, including lung and breast cancer; the safety profile was similar to that observed with carboplatin and paclitaxel alone and promising anti-tumor activity was observed [14–16]. In patients with advanced or metastatic solid tumors, the maximum dose of veliparib was 120 mg BID for 7 days in addition to standard doses of carboplatin and paclitaxel and the RPTD of 100 mg BID was chosen [14]. Higher doses have been cleared in other studies: up to 200 mg BID veliparib with carboplatin and paclitaxel in patients with triple-negative breast cancer [17] and up to 150 mg BID and above with carboplatin, paclitaxel, and bevacizumab in patients with newly diagnosed ovarian cancer (GOG 9923; study ongoing).

In a phase 1 dose-escalation study, partial response (PR) was observed in 11/68 patients (16 %; 2 lung, 2 breast, 2 melanoma, 2 urothelial, head and neck, gastric, unknown primary), complete response (CR) in 2/68 patients (3 %; breast and urothelial), and stable disease in 35/68 patients (51 %). In a phase 2 randomized study of patients with advanced or metastatic NSCLC, the median progression-free survival (PFS) was 5.8 months (95 % CI 4.2–6.1) for patients treated with veliparib plus carboplatin and paclitaxel versus 4.2 months [95 % CI 3.1–5.6; HR 0.71 (95 % CI 0.50–1.13)] for patients treated with placebo plus carboplatin and paclitaxel. Overall survival (OS) was 11.1 months (95 % CI 8.8–13.4) for patients treated with veliparib plus carboplatin and paclitaxel versus 9.1 months (95 % CI 5.4–12.3) for patients treated with placebo plus carboplatin and paclitaxel [16]. Similarly, results from another phase 2 trial (I-SPY 2) demonstrated that for patients with triple-negative breast cancer randomized to veliparib and carboplatin plus standard neoadjuvant therapy, the estimated pathologic complete response rates were 52 versus 26 % for patients treated with standard neoadjuvant therapy alone [15].

The current study evaluated veliparib plus carboplatin and paclitaxel in Japanese patients with NSCLC. The primary objective of this study was determination of the recommended phase 2 dose (RPTD) in a Japanese population; secondary objectives were to assess pharmacokinetics of veliparib, carboplatin, and paclitaxel, and preliminary anti-tumor activity.

## Materials and methods

### Study design

This was an open-label, phase 1 study conducted at a single site in Japan (NCT01617928). The study was conducted in accordance with the International Conference on Harmonization guidelines and the ethical principles of the Declaration of Helsinki. An independent institutional review board approved the study, and all patients provided written informed consent.

### Patients

Eligible adult patients (≥20 years of age) must have a histologically or cytologically confirmed malignant solid tumor, an Eastern Cooperative Oncology Group Performance Status ≤2, and be amenable to standard combination chemotherapy of carboplatin and paclitaxel. Patients must have received ≤1 prior chemotherapy regimen for advanced-stage disease; adjuvant chemotherapy ≥2 years prior to enrollment was not counted as a prior regimen. Other eligibility criteria included normal bone marrow (absolute neutrophil count ≥1500/mcL and platelets ≥150,000/mcL), liver (total bilirubin ≤1.5 × institutional upper limit of normal; AST/SGOT and ALT/SGPT ≤2.5 × institutional upper limit of normal), and kidney (creatinine within upper normal limit of institution’s normal range or creatinine clearance ≥60 mL/min/1.73 m^2^ for patients with creatinine level above institutional normal) function. Men and women of childbearing potential had to agree to use adequate contraception.

Patients were excluded if they had prior treatment with a PARP inhibitor, systemic chemotherapy, or radiotherapy within 3 weeks prior to entering the study (or 6 weeks for nitrosoureas or mitomycin C), or known history of allergic reactions to carboplatin or cremophor-paclitaxel. Other exclusion criteria included toxicities (with the exception of alopecia) from prior systemic chemotherapy, radiation therapy, or sclerotherapy that had not recovered to less than grade 2, uncontrolled intercurrent illness that might impact compliance, peripheral neuropathy (>grade 1), history of seizure disorder, evidence of bleeding diathesis, symptomatic brain metastasis, or hepatitis B surface antigen (HBsAg) positive, HCV antibody positive, or HIV positivity.

### Treatment

All patients received veliparib (40, 80, or 120 mg) BID orally at intervals of approximately 12 h on days 1–7 of each 21-day cycle. The results of previous early phase studies of veliparib in combination with standard carboplatin and paclitaxel informed the doses used in this study; 120 mg BID was pre-specified as the maximum dose based on tolerability in other phase 1 studies in patients with advanced solid tumors [14–16].

Carboplatin (AUC 6 mg/mL min) and paclitaxel (200 mg/m^2^) were given via intravenous (IV) administration on day 3. Six cycles of therapy were planned. Patients could receive standard supportive care including treatment with a pre-medication regimen to reduce severity of hypersensitivity reactions according to the paclitaxel prescribing information [18].

Platelets had to be ≥100,000/mcL, and absolute neutrophil count had to be ≥1500/mcL before initiation of the next cycle of therapy. Treatment could be postponed for up to 21 days because of toxicity; longer delays led to discontinuation. A delay in any one component of the regimen required delay of all drugs within the regimen.

### Assessments

A complete medical history was collected during the screening visit, including detailed oncology history. A physical examination was performed at all visits (screening, days 1, 3, 8, and 15).

The safety of veliparib in combination with carboplatin and paclitaxel in Japanese patients was assessed by the evaluation of study drug exposure, adverse events (AEs), serious AEs (SAEs), deaths, laboratory profiles, physical examination, and vital signs. All patients who received at least one dose of veliparib were included in the safety analysis. Treatment-emergent AEs were summarized by system organ class and preferred term according to the medical dictionary for regulatory activities (MedDRA) AE coding dictionary and were graded according to the NCI CTCAE version 4.03 [19]. AEs were assessed for severity and relationship with veliparib.

The study followed a dose-escalation scheme whereby at least three patients were enrolled to each dose level in a stepwise manner, starting with 40 mg. The dose-limiting toxicity (DLT) evaluation period was cycle 1. Events were considered as DLTs if they were considered associated with veliparib (possibly and probably related) and met the following criteria: any grade 4 neutrophil lasting longer than 7 days, any grade 4 thrombocytopenia (platelet ≤25,000/mcL), febrile neutropenia (temperature ≥38.3 or ≥38 °C sustained over a 1-h period associated with absolute neutrophil count of <0.5 × 10^9^/L), any event resulting in delay of the next treatment cycle by more than 3 weeks due to toxicity, or any other ≥grade 3 non-hematological toxicity that represented at least a two-grade increase from baseline and was clinically significant or symptomatic. Other toxicities that did not meet the above criteria may be considered as a DLT if the toxicities were considered to be associated with study treatment and clinically significant. Overall toxicity was considered in dose-escalation decisions.

A CT scan of the full chest and abdomen with images of the liver and adrenal glands was performed for all tumor assessments at screening (within 3 weeks prior to registration) and at the end of every 2 cycles (6 weeks). Tumor assessments were performed within 7 days of the scheduled date. Patients who had at least one measurable lesion at baseline and who received at least one dose of veliparib were evaluated for anti-tumor response criteria using RECIST version 1.1 [20].

Blood samples were collected on days 1 and 3 of cycle 1 for veliparib pharmacokinetic analysis and on day 3 of cycle 1 for paclitaxel and carboplatin pharmacokinetic analysis. On days 1 and 3 of cycle 1, blood was collected to assay veliparib concentrations before the morning dose of veliparib and 0.5, 1, 1.5, 2, 4, 6, and 8 h after dosing. On day 3 of cycle 1, blood was collected to assay paclitaxel concentrations before administration of the morning dose of paclitaxel and 1, 2, 3 (immediately after ending paclitaxel infusion), 4, 6, 8, and 24 h after dosing. On day 3 of cycle 1, blood was collected to assay carboplatin concentrations immediately after ending paclitaxel infusion (before beginning carboplatin infusion) and 0.25, 1, 3, 5, and 21 h after dosing. On day 3 of cycle 1, veliparib was ingested at essentially the same time as paclitaxel infusion began and carboplatin infusion immediately followed paclitaxel infusion. Plasma samples were stored at −20 °C until shipment to AbbVie. Plasma concentrations of veliparib and paclitaxel were determined using validated liquid chromatography methods with tandem mass spectrometric detection (LCMS/MS). Plasma concentrations of carboplatin were determined using an inductively coupled plasma mass spectrometry. Standard pharmacokinetic parameters were determined using non-compartmental methods.

## Results

### Patients and treatment

Twelve patients were enrolled (May 2012–January 2013) and received at least one dose of veliparib. All enrolled patients had advanced or metastatic non-small cell lung cancer (NSCLC). Patient characteristics are summarized in Table 1.

**Table 1:** Patient characteristics (safety population)

Characteristic	Number (%) of patients			
	Veliparib 40 mg BID *n* = 3	Veliparib 80 mg BID *n* = 3	Veliparib 120 mg BID *n* = 6	Total *N* = 12
Sex, *n* (%)				
Female	0	0	2	2 (17)
Male	3	3	4	10 (83)
Age, years, median (range)	71 (67–72)	56 (44–60)	67 (47–73)	67 (44–73)
Race, *n* (%)				
Japanese	3	3	6	12 (100)
Median duration of disease^a^ (range), days	63 (22–84)	70 (46–77)	62.5 (48–2739)	64 (22–2739)
Histology at time of diagnosis, *n* (%)				
Adenocarcinoma	1	3	6	10 (83)
Other	1	0	0	1 (8)
Not available	1	0	0	1 (8)
ECOG performance status^b^, *n* (%)				
0	1	2	3	6 (50)
1	2	1	3	6 (50)
Clinical stage^c^, *n* (%)				
Stage IIIB	0	0	1	1 (8)
Stage IV	2	3	4	9 (75)
Postoperative recurrence	1	0	1	2 (17)
Tumor burden^b^, *n* (%)				
Locally advanced	1	0	1	2 (17)
Metastatic	2	3	5	10 (83)
Tobacco, *n* (%)				
Current	0	0	2	2 (17)
Former	3	3	2	8 (67)
Never used	0	0	2	2 (17)
Prior oncology surgery	1	0	1	2 (17)
Prior systemic or radiation therapy	0	0	0	0 (0)

*ECOG* Eastern Cooperative Oncology Group

^a^Days from date of diagnosis to date of first dose of study drug

^b^Baseline

^c^At time of enrollment

### Safety

Veliparib plus carboplatin and paclitaxel was well tolerated in this population of Japanese patients with NSCLC. AEs were consistent with toxicities commonly associated with the combination. Treatment-emergent AEs that occurred in more than 20 % of patients are summarized in Table 2. The majority of AEs were mild to moderate (grade 1 and 2). The most commonly occurring AEs of any grade (without attribution to veliparib) included leukopenia (*n* = 12; 100 %), neutropenia (*n* = 12; 100 %), arthralgia (*n* = 11; 92 %), myalgia (*n* = 10; 83 %), anemia (*n* = 10; 83 %), and thrombocytopenia (*n* = 9; 75 %). The most commonly occurring grade 3/4 AEs (without attribution to veliparib) included neutropenia (*n* = 12; 100 %), leukopenia (*n* = 4; 33 %), and anemia (*n* = 3; 25 %). Although hematological toxicities were commonly observed, these toxicities were manageable with medication or dose reductions and delays.

**Table 2:** Treatment-emergent adverse events

AEs in ≥20% of all patients, *n*	Number (%) of patients									
	Veliparib 40 mg BID *n* = 3		Veliparib 80 mg BID *n* = 3		Veliparib 120 mg BID *n* = 6		Total any grade *N* = 12		Total grade ¾ *N* = 12	
	All grades	Grade 3/4	All grades	Grade 3/4	All grades	Grade 3/4	All grades	At least possibly related	All grades	At least possibly related
Any AE	3	3	3	3	6	5	12	11	12	11
Blood and lymphatic system disorders										
Anemia	3	1	1	0	6	2	10	9	3	3
Leukopenia	3	0	3	0	6	4	12	11	4	3
Neutropenia	3	3	3	3	6	6	12	11	12	11
Thrombocytopenia	2	0	1	0	6	0	9	8	0	0
Gastrointestinal disorders										
Constipation	3	0	0	0	3	0	6	4	0	0
Diarrhea	1	0	0	0	2	0	3	2	0	0
Nausea	2	0	2	0	4	0	8	5	0	0
General disorders, admin site conditions										
Fatigue	2	0	0	0	3	0	5	4	0	0
Laboratory investigations										
Increased alanine aminotransferase	2	0	2	0	4	0	8	8	0	0
Increased aspartate aminotransferase	2	0	2	0	4	0	8	8	0	0
Metabolism and nutrition disorders										
Decreased appetite	2	0	1	0	5	0	8	6	0	0
Hypoalbuminemia	1	0	0	0	2	0	3	3	0	0
Hyponatremia	1	1	0	0	2	1	3	1	2	0
Musculoskeletal and connective tissue										
Arthralgia	3	0	2	0	6	0	11	2	0	0
Myalgia	3	0	3	0	4	0	10	1	0	0
Nervous system disorders										
Peripheral neuropathy	1	0	1	0	2	0	4	1	0	0
Peripheral sensory neuropathy	1	0	0	0	4	1	5	1	1	1
Skin and subcutaneous tissue disorders										
Alopecia	1	0	2	0	5	0	8	2	0	0
Rash	1	0	3	0	0	0	4	4	0	0
Vascular disorders										
Hypertension	0	0	2	1	0	0	2	2	1	1

The median number of cycles was 4 (range 1–6). No treatment-emergent AEs led to interruption of veliparib, carboplatin, or paclitaxel. Three patients (25 %) experienced a treatment-emergent AE that resulted in dose reductions in veliparib (*n* = 1 grade 3 hypertension, 80 mg BID and *n* = 2 grade 3 anemia, 120 mg BID). Seven patients (58 %) experienced a treatment-emergent AE that led to dose delays in veliparib, carboplatin, or paclitaxel. Two patients experienced an AE of peripheral sensory neuropathy that led to discontinuation (*n* = 1 grade 2 event beginning on day 79 and continuing as of day 100 and *n* = 1 grade 3 event beginning on day 48 and continuing as of day 78). There were no SAEs or AEs that led to death. There were no DLTs at any dose level during the DLT assessment period. There were no clinically relevant changes in laboratory chemistries, urinalysis, or vital signs. The RPTD of veliparib administered with carboplatin and paclitaxel was determined to be 120 mg BID.

### Pharmacokinetics

Pharmacokinetic parameters are summarized in Table 3. Veliparib *C*_max_ and AUC values were approximately dose proportional at the three dose levels of veliparib (40, 80, and 120 mg). For each dose of veliparib, maximum plasma veliparib concentrations were observed approximately 2–3 h after administration. Co-administration of carboplatin and paclitaxel had no significant effect on veliparib *T*_max_, dose-normalized *C*_max_, or dose-normalized AUC (*p* ≥ 0.2377; Table 3). Carboplatin and paclitaxel pharmacokinetics were comparable when co-administered with 40, 80, or 120 mg veliparib BID, respectively, showing no evidence of an effect of veliparib on carboplatin or paclitaxel pharmacokinetics (Table 3).

**Table 3:** Mean ± SD pharmacokinetic parameters after administration of veliparib with and without carboplatin + paclitaxel

Veliparib PK parameters (units)	Veliparib dose level			All patients *N* = 12
	40 mg BID *n* = 3	80 mg BID *n* = 3	120 mg (BID) *n* = 6	
Study day 1 (after veliparib alone)^a^				
*T*_max_ (h)	2.5 ± 1.3	3.3 ± 1.2	3.0 ± 1.1	3.0 ± 1.1
*C*_max_ (ng/mL)	327 ± 47.7	481 ± 27.8	844 ± 152	–
AUC_0–8_ (ng h/mL)	1287 ± 370	2137 ± 197	3993 ± 796	–
AUC_∞_ (ng h/mL)	1838 ± 671	2804 ± 266	5757 ± 1166	–
*C*_max_/dose, (ng/mL)/mg	8.18 ± 1.19	6.01 ± 0.35	7.03 ± 1.27	7.06 ± 1.29
AUC_0–8_/dose (ng h/mL)/mg	32.2 ± 9.24	26.7 ± 2.46	33.3 ± 6.34	31.4 ± 6.69
AUC_∞_/dose (ng h/mL)/mg	46.0 ± 16.8	35.0 ± 3.33	48.0 ± 9.72	44.2 ± 11.3
Study day 3 (after carboplatin + paclitaxel)^a^				
*T*_max_ (h)	2.5 ± 1.3	4.0 ± 0.0	1.4 ± 0.5	2.3 ± 1.3
*C*_max_ (ng/mL)	345 ± 149	400 ± 48.8	1245 ± 149	–
AUC_0–8_ (ng h/mL)	1511 ± 457	2157 ± 48.3	5007 ± 960	–
AUC_0–12_ (ng h/mL)	1929 ± 572	2781 ± 94.5	6246 ± 1054	–
*C*_max_/dose, (ng/mL)/mg	8.63 ± 3.73	5.00 ± 0.61	10.4 ± 1.24	8.60 ± 2.92
AUC_0–8_/dose (ng h/mL)/mg	37.8 ± 11.4	27.0 ± 0.60	41.7 ± 8.00	37.0 ± 9.63
AUC_0–12_/dose (ng h/mL)/mg	48.2 ± 14.3	34.8 ± 1.18	52.0 ± 8.78	46.8 ± 11.3
Carboplatin PK parameters (units)				
*T*_max_ (h)	1.0 ± 0.0	1.0 ± 0.0	1.0 ± 0.0	1.0 ± 0.0
*C*_max_ (µg/mL)	22.3 ± 2.37	24.3 ± 1.16	22.0 ± 2.45	22.6 ± 2.25
AUC∞ (µg h/mL)	105 ± 12.3	105 ± 7.14	100 ± 5.73	103 ± 7.67
AUC_t_ (µg h/mL)	94.1 ± 9.91	95.1 ± 5.83	90.3 ± 5.45	92.5 ± 6.54
Paclitaxel PK parameters (units)				
*T*_max_ (h)	3.1 ± 0.2	3.0 ± 0.0	2.7 ± 0.4	2.9 ± 0.3
*C*_max_ (µg/mL)	7.56 ± 0.48	6.40 ± 1.22	6.69 ± 1.43	6.84 ± 1.20
AUC∞ (µg h/mL)	27.2 ± 0.23	22.8 ± 2.37	25.9 ± 3.16	25.5 ± 2.89
AUC_t_ (µg h/mL)	26.4 ± 0.30	22.1 ± 2.44	25.3 ± 3.09	24.8 ± 2.86

^a^Veliparib administered orally BID on days 1 through 7; carboplatin (AUC 6 mg/mL min) and paclitaxel (200 mg/m^2^) administered intravenously on day 3. Based on carboplatin concentrations, the last sampling time point was 21 h after starting intravenous infusion of carboplatin

### Efficacy

**Fig. 1:**
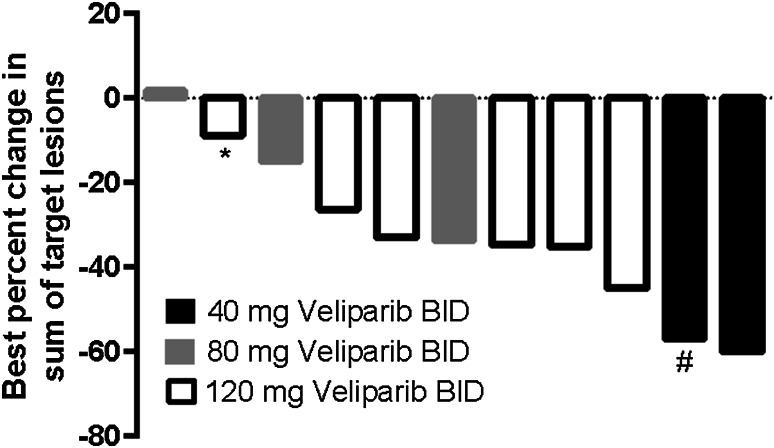
Greatest percent decrease from baseline in the sum of tumor sizes of target lesions assessed by investigator (efficacy population). For 9 patients, the greatest percent decrease from baseline in the sum of target lesions occurred following completion of 6 cycles of the combination regimen. One patient (40 mg BID) achieved the greatest percent decrease from baseline in cycle 4 (indicated by #). One patient (120 mg BID) achieved the greatest percent decrease from baseline in cycle 2 (indicated by *)

**Fig. 2:**
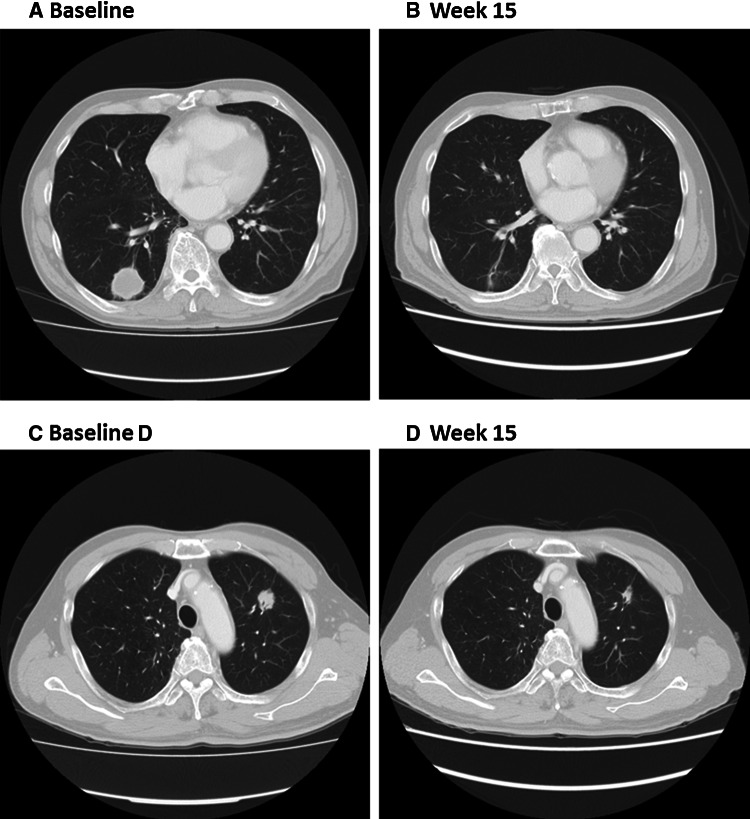
Partial response to veliparib 40 mg BID and carboplatin and paclitaxel. CT images at baseline (**a**, **c**) and following treatment (15 weeks; **b**, **d**) in an approximately 70-year-old male with advanced NSCLC

Eleven patients had at least one measurable lesion at baseline and comprised the efficacy analysis population. The overall response rate was 55 % (6/11 patients, 95 % CI 23.4–83.3 %). Six patients achieved a PR (*n* = 2, 40 mg BID and *n* = 4, 120 mg BID); of these, 3 achieved PR at the first CT scan. Four patients achieved stable disease (SD) (*n* = 2, 80 mg BID and *n* = 2, 120 mg BID). One (80 mg BID) developed progressive disease (PD). The median PFS was 92 days (range 21–143). The best percent change from baseline in the sum of target lesions is presented in Fig. 1. The greatest percent decrease from baseline in the sum of target lesions occurred in cycle 4 for 1 patient (40 mg BID) and in cycle 2 for 1 patient (120 mg BID). For all other patients, the greatest percent decrease from baseline in the sum of target lesions occurred following completion of the combination regimen. Figure 2 shows a CT image at baseline and following treatment in a patient with advanced NSCLC who achieved a PR following treatment (40 mg BID).

## Discussion

The current study established the safety and tolerability of veliparib up to 120 mg BID in combination with carboplatin AUC 6 and paclitaxel 200 mg/m^2^ every 3 weeks in Japanese patients with NSCLC. The AEs that occurred were similar to those expected with the carboplatin and paclitaxel regimen alone [21]. Hematological toxicities were common and included neutropenia, anemia, thrombocytopenia, and leukopenia. These toxicities were generally managed with medication or dose reductions or delays. The RPTD of veliparib in combination with carboplatin and paclitaxel was determined to be 120 mg BID.

The doses used in the current study were expected to be biologically active. Results of a previous phase 0 study showed substantial inhibition of PARP activity in tumor biopsies collected 3–6 h after dosing in 3 subjects who received a single dose of 25 mg veliparib (92, 95, and 100 %) [22]. Complete inhibition of PARP activity in PBMCs was achieved and maintained for 24 h in 3 of those who received 50 mg veliparib. For the 50-mg group, PARP inhibition in tumor biopsies averaged 75 % 3–6 h after dosing (*N* = 3) and 74 % 24 h after dosing (*N* = 3). Therefore, both 25 mg and 50 mg veliparib were found to be biologically active doses [22]; thus, the doses of 40 mg BID or greater used in the phase 1 study could contribute to observed activity.

As expected, administration of carboplatin and paclitaxel in combination with veliparib did not impact the pharmacokinetic profile of veliparib. Veliparib is a highly soluble and permeable compound. It is primarily eliminated by renal excretion and to a lesser extent by multiple cytochrome P450 enzymes in humans and is not an inhibitor of major cytochrome P450 enzymes or major drug transporters at therapeutic doses [23]. Veliparib pharmacokinetic parameters were approximately dose proportional at the dose levels evaluated (40, 80, 120 mg BID). Effects of veliparib on carboplatin and paclitaxel were evaluated indirectly by comparing the carboplatin or paclitaxel pharmacokinetic profiles following co-administration veliparib (40, 80, or 120 mg BID). Although the comparisons do not provide definitive evidence, the findings were consistent with expectation of no pharmacokinetic interaction between veliparib and carboplatin or paclitaxel. Consistent with the physicochemical property and the elimination pathways of veliparib, the pharmacokinetic profile of veliparib also was generally comparable between Japanese and Western populations.

The sample size of the current study was sufficient to investigate pharmacokinetics and identify an appropriate RPTD. Patients with histologically or cytologically confirmed malignant solid tumors were eligible for this study as long as they received only ≤1 prior chemotherapy regimen. Despite these selection criteria, all of the patients enrolled in the current study were patients with NSCLC. The overall response rate was 55 %. As the primary objectives were safety and pharmacokinetic assessment, tumor assessment data were not collected after completing study treatment. As a result, only 4 subjects had a final tumor assessment with PD and the remaining 8 subjects were censored without documented PD; the estimated median PFS is 92 days.

PARP inhibitors are believed to be particularly effective in tumors with defects in homologous recombination such as those with BRCA mutation. The results of this trial suggest that veliparib may be effective and improve sensitivity to chemotherapy in tumors without identified defects in DNA repair, including NSCLC. In addition, in a recently reported placebo-controlled phase 2 trial in advanced NSCLC, a trend toward improved PFS and OS was observed when veliparib was added to carboplatin and paclitaxel [16]. Veliparib has also shown preliminary efficacy in other solid tumor types, including triple-negative breast, head and neck, and ovarian cancers [14, 17]. Given the limited heterogeneity in this phase 1 study, further study in other solid tumor types is warranted. Future larger-scale studies will be useful to verify efficacy in these tumor types.

## Conclusions

The current study established tolerability of the addition of veliparib to platinum-based chemotherapy. The RPTD of veliparib administered with carboplatin and paclitaxel in Japanese patients was determined to be 120 mg BID. An overall response rate of 55 % warrants future studies. Future studies are examining veliparib at this dose, alone and in combination with carboplatin and paclitaxel, in patients with advanced NSCLC and other solid tumors.

## Abbreviations

AE: Adverse event
ALT: Alanine transaminase
AST: Aspartate transaminase
AUC: Area under the curve
BID: Bis in die; two times a day
CI: Confidence interval
CT: Computed tomography
DNA: Deoxyribonucleic acid
DLT: Dose-limiting toxicity
EGFR: Epidermal growth factor receptor
GOG: Gynecologic Oncology Group
HBsAg: Hepatitis B surface antigen
HCV: Hepatitis C virus
HIV: Human immunodeficiency virus
HR: Hazard ratio
LCMS: Liquid chromatography mass spectrometry
MedDRA: Medical dictionary for regulatory activities
NSCLC: Non-small cell lung cancer
OS: Overall survival
PARP: Poly (ADP-ribose) polymerase
PBMC: Peripheral blood mononuclear cell
PD-1: Programmed cell death protein 1
PD: Progressive disease
PFS: Progression-free survival
PK: Pharmacokinetic
PR: Partial response
RPTD: Recommended phase 2 dose
SAE: Serious adverse event
SGOT: Serum glutamic oxaloacetic transaminase
SGPT: Serum glutamic pyruvic transaminase
VEGF: Vascular endothelial growth factor receptor
